# Autophagy Receptor Protein Tax1-Binding Protein 1/TRAF6-Binding Protein Is a Cellular Substrate of Enteroviral Proteinase

**DOI:** 10.3389/fmicb.2021.647410

**Published:** 2021-06-04

**Authors:** Yasir Mohamud, Yuan Chao Xue, Huitao Liu, Chen Seng Ng, Amirhossein Bahreyni, Honglin Luo

**Affiliations:** ^1^Centre for Heart Lung Innovation, St. Paul’s Hospital, Vancouver, BC, Canada; ^2^Department of Pathology and Laboratory Medicine, University of British Columbia, Vancouver, BC, Canada; ^3^Department of Experimental Medicine, University of British Columbia, Vancouver, BC, Canada

**Keywords:** enterovirus, coxsackievirus, selective autophagy, TRAF6-binding protein, Tax1-binding protein 1, optineurin, calcium-binding and coiled-coil domain-containing protein 2/nuclear dot 10 protein 52

## Abstract

Enteroviruses (EVs) usurp the host autophagy pathway for pro-viral functions; however, the consequence of EV-induced diversion of autophagy on organelle quality control is poorly defined. Using coxsackievirus B3 (CVB3) as a model EV, we explored the interplay between EV infection and selective autophagy receptors, i.e., Tax1-binding protein 1/TRAF6-binding protein (T6BP), optineurin (OPTN), and nuclear dot 10 protein 52 (NDP52), known to be involved in regulating the clearance of damaged mitochondria, a process termed as mitophagy. Following CVB3 infection, we showed significant perturbations of the mitochondrial network coincident with degradation of the autophagy receptor protein T6BP, similar phenomenon to what we previously observed on NDP52. Notably, protein levels of OPTN are not altered during early infection and slightly reduced upon late infection. Cell culture studies revealed that T6BP degradation occurs independent of the function of host caspases and viral proteinase 3C, but requires the proteolytic activity of viral proteinase 2A. Further investigation identified the cleavage site on T6BP after the amino acid 621 that separates the C-terminal ubiquitin-binding domain from the other functional domains at the N-terminus. Genetic silencing of T6BP and OPTN results in the attenuation of CVB3 replication, suggesting a pro-viral activity for these two proteins. Finally, functional assessment of cleaved fragments from NDP52 and T6BP revealed abnormal binding affinity and impaired capacity to be recruited to depolarized mitochondria. Collectively, these results suggest that CVB3 targets autophagy receptors to impair selective autophagy.

## Introduction

Enteroviruses (EVs) are associated with diverse human diseases and continue to pose a global health threat. Recent outbreaks of EV-D68 and EV-A71 in North America and Southeast Asia, respectively, have raised concerns, particularly in the young and immune-compromised populations (Yang et al., 2009; Wu et al., 2010; Messacar et al., 2016). The threat of global outbreaks coupled with a lack of FDA-approved effective antivirals/vaccines against non-polio EVs warrants continued research efforts.

Enteroviruses have evolved to usurp the cellular recycling machinery of autophagy, a catabolic process that normally acts to degrade large protein aggregates, damaged cellular organelles, and invading microbes, including viruses. Although autophagy may participate in the bulk and non-selective degradation of cellular cargo in times of nutrient stress (Mizushima, 2007), more targeted clearance and recycling of cellular cargo can take place, particularly of damaged organelles, such as the endoplasmic reticulum, mitochondria, peroxisomes, and lysosomes (Anding and Baehrecke, 2017). With respect to mitochondria, quality control processes are especially vital in mitochondria-rich tissues, such as heart, brain, and muscles, where the accumulation of damaged mitochondria can result in permanent damage and/or cellular death. The selective autophagic degradation of damaged mitochondria, coined as mitophagy (Lemasters, 2005), is a regulated process that is initiated by the serine/threonine kinase PTEN-induced kinase 1 (PINK1). Accumulated PINK1 on the surface of depolarized mitochondria can phosphorylate serine 65 of ubiquitin (Kane et al., 2014; Koyano et al., 2014), resulting in the recruitment and activation of the E3 ubiquitin ligase Parkin (Geisler et al., 2010; Vives-Bauza et al., 2010). Subsequently, ubiquitin-modified mitochondrial proteins are recognized by selective autophagy receptors that function to recruit damaged mitochondria to autophagic platforms for clearance.

Autophagy receptor proteins play essential roles in the selective clearance of various cellular cargos, including dysfunctional mitochondria (Johansen and Lamark, 2011). The mitophagy process in particular relies on specific receptor proteins, such as optineurin (OPTN), calcium-binding and coiled-coil domain-containing protein 2 (CALCOCO2)/nuclear dot 10 protein 52 (NDP52), and CALCOCO3/Tax1-binding protein 1 (TAX1BP1)/TRAF6-binding protein (T6BP; Lazarou et al., 2015). These proteins share similar domain structure, each harboring either single or multiple copies of ubiquitin-associated domain(s) (e.g., UBA or UBZ) as well as autophagy-targeting LC3 interaction regions (LIR). In addition to mitochondrial quality control, autophagy receptors also play a role in the regulation of innate immunity. The TANK-binding kinase 1 (TBK1) is a master regulator of type I interferon (IFN) signaling and also a key upstream kinase for autophagy receptors, NDP52, OPTN, and T6BP (Richter et al., 2016). NDP52 acts to quench persistent IFN signaling in part by targeting the mitochondrial antiviral signaling (MAVS) protein for autophagic degradation (Jin et al., 2017). Negative regulation of antiviral IFN signaling was also reported for OPTN in response to RNA virus (Mankouri et al., 2010). Moreover, a similar inhibitory role was demonstrated for T6BP in response to NF-κB-mediated cytokine signaling (Morriswood et al., 2007).

We previously uncovered that EV-encoded proteinases target several autophagy receptor proteins, including sequestosome 1/p62, neighbor of BRCA1 (NBR1), and NDP52 to evade the host’s effort in degrading viral particles and its components, termed as virophagy (Shi et al., 2013, 2014; Mohamud et al., 2019). However, it remains to be seen whether other receptor proteins can be targeted by virus, and additionally, whether viral cleavage of autophagy receptors has deleterious consequences for host quality control processes, such as mitophagy. In the current study, we identified T6BP as a novel substrate of coxsackievirus B3 (CVB3) proteinase and further elucidated the consequence of T6BP and NDP52 cleavage in selective autophagy.

## Materials And Methods

### Cell Culture, Viral Infection, and Chemicals

HeLa cells (American Type Culture Collection) were cultured in Dulbecco’s modified Eagle’s medium supplemented with 10% fetal bovine serum and a penicillin/streptomycin cocktail (100 μg/ml). For induction of type I IFN, the synthetic analog of double-stranded RNA, poly-inosinic-cytidylic acid-high molecular weight (poly I:C-HMW, InvivoGen, #tlrl-pic), was used. For CVB3 infection, cells were either sham-infected with PBS or inoculated with CVB3 (Kandolf strain) at different multiplicity of infection (MOI) as specified in the figure legends.

### Plasmids and siRNA

The GFP-T6BP plasmid was generated by subcloning T6BP ORF (accession #: BC050358.2) into pEGFP-C1 vector at restriction sites (BspE1 and KpnI). Mutant T6BP constructs (G715E, G668E, G636E, G522E, G687E, and G621E) were generated using custom gBLOCKS (IDT), and mutant fragments were cloned through restriction digestion with either EcoRI/KpnI or BglII/EcoRI. Cloning of truncated T6BP-ΔUBZ (Δ687-789) was performed using Kpn2I/KpnI restriction sites with the following primers (forward primer: AAA TTT TCC GGA ATG ACA TCC TTT CAA GAA GTC CCA TT; reverse primer: AAA TTT GGT ACC CTA ATC AGG CCG ACT AAA GTT TC). The single guide RNA (sgRNA) sequence targeting human *T6BP* is CATGTCATCTTTCAAAATG (Exon 2) and was cloned into pSpCas9-2A-GFP vector. The scrambled small interfering RNA (siRNA; sc-37007) and siRNAs targeting NDP52 (sc-93738), OPTN (sc-39054), and T6BP (sc-106831) were purchased from Santa Cruz Biotechnology. For transfection, cells were transiently transfected with plasmid cDNAs or sgRNAs using Lipofectamine 2000 (Invitrogen, 11668-019) following the manufacturer’s instructions.

### Purification of CVB3 2A^pro^

pET-28a plasmids encoding wild-type (WT) CVB3 2A^pro^ were transformed into C41 (DE3) *Escherichia coli* and then plated onto kanamycin (50 μg/ml) agar plates. A starter culture from a single colony was grown overnight and then diluted 100-fold in Terrific Broth (Sigma, T9179). Expression was induced with 1 mm isopropyl β-D-1-thiogalactopyranoside (IPTG) after cultures reached an OD600 of 0.6–0.8 and proceeded at 25°C for 5 additional hours. Protein was purified using Ni-NTA Fast Start (Qiagen, 30600) according to the manufacturer’s instructions. Catalytically inactive 2A^mut^ (C109A) was generated as previously described (Jagdeo et al., 2015).

### *In vitro* Cleavage Assay

*In vitro* cleavage assay was performed as previously described (Mohamud et al., 2019). Briefly, HeLa lysates (20 μg) were incubated with WT or catalytically inactive (C109A) CVB3-2A (0.1 μg) in cleavage assay buffer (20 mm HEPES pH 7.4, 150 mm KOAc, and 1 mm DTT) for the indicated times at 37°C. Reactions were terminated with 6× sample buffer and subjected to Western blot analysis.

### Western Blot Analysis

Cells were lysed in buffer (10 mm HEPES pH 7.4, 50 mm Na pyrophosphate, 50 mm NaF, 50 mm NaCl, 5 mm EDTA, 5 mm EGTA, 100 μm Na_3_VO_4_, and 0.1% Triton X-100), and Western blotting was conducted using the following primary antibodies: T6BP (Santa Cruz Biotechnology, sc-393143), β-actin (ACTB, Sigma-Aldrich, A5316), cleaved caspase 3 (CST, #9661), EIF4G (CST, C45A4), Flag (Sigma, F1804), GFP (Life Technologies, A-6455), anti-myc (Upstate, 06-549), anti-HA (Roche, #11867423001), NUP98 (CST, C39A3), OPTN (Proteintech, 10837-1-AP), and VP1 (Dako, M706401-1).

### Immunoprecipitation

Immunoprecipitation of GFP-tagged T6BP construct was performed using GFP ABfinity recombinant monoclonal antibody (Invitrogen, G10362) according to the manufacturer’s instructions. In brief, HeLa cell lysates were incubated with anti-GFP antibody at 4°C for 3 h, followed by 1 h incubation with Pierce Protein A/G magnetic beads (#88802). Immunoprecipitation of Flag-tagged construct was performed with EZView Red Anti-FLAG M2 Affinity Gel (Sigma, F2426) following the manufacturer’s guidelines. After three washes, the bound proteins were eluted with 2× SDS sample buffer and then subjected to Western blot analysis.

### Real-Time Quantitative RT-PCR

Total RNA was extracted using the RNeasy Mini kit (Qiagen, 74104). To determine the mRNA level of IFN-β, qPCR targeting IFNβ (forward primer: GTC TCC AAA TTG CTC TC; reverse primer: ACA GGA GCT TCT GAC ACT GA) was conducted in a 10 μl reaction containing 500 ng of RNA using the Luna^®^ Universal One-Step RT-qPCR kit according to the manufacturer’s instructions. The results were normalized to GAPDH mRNA. The PCR reaction was performed on a QuantStudio 6 Pro (Applied Biosystems). Samples were run in triplicate and analyzed using comparative CT (2^−ΔΔCT^) method with control samples and presented as relative quantitation (RQ).

### Confocal Microscopy

HeLa cells were cultured in eight-well chamber slides (Labtek, 155411) for 24 h prior to treatment. Cell membrane integrity was assessed with the amine-reactive dye LIVE/DEAD (L34958; Thermo Fisher Scientific) according to the manufacturer’s instructions. After fixation in 4% paraformaldehyde, cells were washed thrice in PBS and mounted with FluoroShield with 4,6-diamidino-2-phenylindole (Sigma-Aldrich, F6057). Images were captured with the Zeiss LSM 880 Inverted Confocal Microscopy using a 63× objective lens. Co-localization was assessed using Pearson’s correlation. Images were analyzed on ImageJ 1.53C NIH software for *n* ≥ 30 cells and presented as mean Rr ± standard deviation (SD). Mitochondrial area was measured using ImageJ Mito-Morphology Macro and normalized to total cellular area as described (Dagda et al., 2009). Mitochondrial network branching was quantified using Mitochondrial Network Analysis (MiNA) software as previously described (Valente et al., 2017).

### Viral Titer Measurement

Samples were serially diluted and overlaid on 60-well Terasaki plates of HeLa cells. After 48 h incubation, 50% tissue culture infective dose titer (TCID50) was calculated by the statistical method of Reed and Muench (1938). Titers were expressed as plaque forming unit/ml with one infectious unit equal to 0.7 TCID50 as described previously (Davis et al., 1972).

### Statistical Analysis

Results are presented as mean ± SD. Statistical analysis was performed with unpaired Student’s *t*-test or ANOVA. A *p* < 0.05 was considered to be statistically significant. All results presented are representative of at least three independent experiments.

## Results

### CVB3 Infection Disrupts Mitochondrial Network

**Figure 1:**
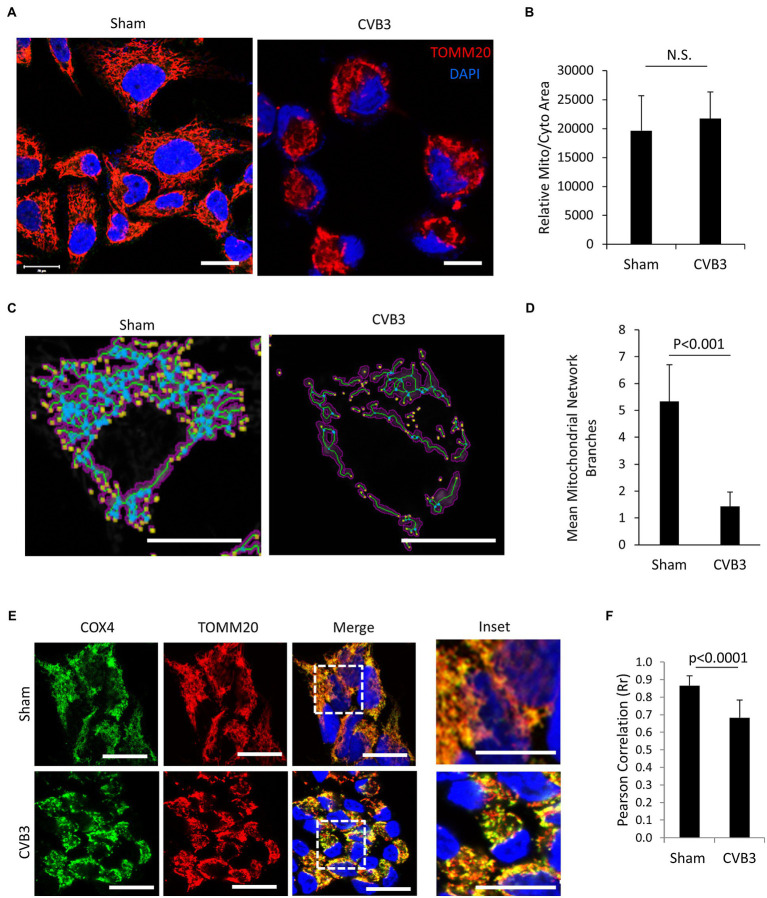
CVB3 infection disrupts mitochondrial network. (**A** and **C**) HeLa cells were infected with CVB3 (MOI = 10, 5 h) or PBS (sham). Cells were fixed and immunostained with anti-TOMM20 (red) and counterstained with 4,6-diamidino-2-phenylindole (DAPI; blue). Mitochondrial network was analyzed using MiNA tool **(C)**. Magenta, binarized mitochondrial area; green, morphological skeleton; yellow, endpoints; and blue, junctions. Scale bar = 20 μm. (**B** and **D**) Mitochondrial area normalized to total cellular area was measured using NIH ImageJ and presented as mean ± SD, *n* = 30 cells **(B)**. The mitochondrial network branches were quantified using the MiNA tool and presented as mean ± SD, *n* = 30 cells **(D)**. **(E)** HeLa cells were infected with CVB3 (MOI = 10, 5 h) or PBS (sham). Cells were fixed and immunostained with anti-COX4 (green) and anti-TOMM20 (red), followed by counterstain with DAPI (blue). Scale bar = 20 μm. **(F)** Pearson’s correlation (Rr) of COX4 and TOMM20 following sham or CVB3 infection was quantified from **(C)** and presented as mean ± SD, *n* = 30 cells.

Enteroviruses have been shown to hijack the host autophagy pathway for efficient replication, but the consequences of diverting cellular autophagy toward pro-viral function are not completely understood. Mitochondria are organelle substrates of autophagy that require efficient recycling to maintain optimal cell function and regulate inflammatory and apoptotic signaling (Wang and Youle, 2009). To investigate EV-induced pathogenesis, we infected HeLa cells with CVB3 at a MOI of 10 and monitored the mitochondrial network using the translocase of outer mitochondrial membrane 20 (TOMM20). Upon CVB3 infection, the mitochondrial network was severely collapsed and demonstrated a fragmented appearance (Figure 1A). Despite significant alterations in the mitochondrial network, the mitochondrial area remained unchanged when normalized to total cellular area (Figure 1B), likely reflecting the coincident appearance of mitochondrial disruption with cytopathic effects. To further assess the disruption of mitochondrial network, we utilized the MiNA tool (Valente et al., 2017) in sham- and CVB3-infected cells. The results revealed that CVB3-infected cells exhibited significantly reduced branching of mitochondrial network (Figures 1C,D). The mitochondrial inner membrane is the site of cellular respiration and houses the electron transport chain, including cytochrome C oxidase subunit 4 (COX4), a component of complex 4. In sham-infected control cells, COX4 significantly overlapped with the outer mitochondrial membrane marker TOMM20 (Rr = 0.87 ± 0.06); however, following CVB3 infection, a significant decrease was observed in the co-localization of COX4 with TOMM20 (Rr = 0.68 ± 0.10), indicating a disruption of mitochondrial integrity (Figures 1E,F).

### CVB3 Targets Selective Autophagy Receptor T6BP but Not OPTN

**Figure 2:**
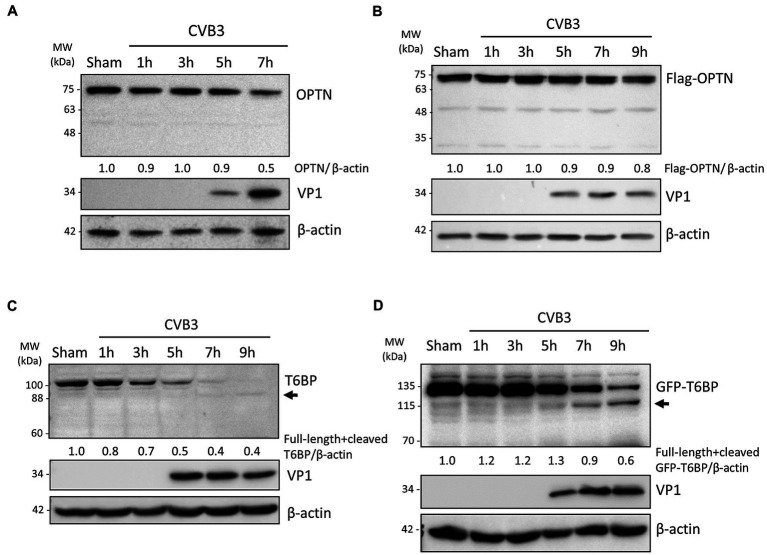
CVB3 targets selective autophagy receptor T6BP but not OPTN. (**A** and **C**) HeLa cells were sham- or CVB3-infected (MOI = 10) for 1 h, 3 h, 5 h, 7 h, or 9 h as indicated. Cell lysates were harvested and probed with anti-OPTN **(A)** or anti-T6BP **(C)**. Protein levels of OPTN and both full-length and cleaved T6BP were quantified by densitometric analysis using NIH ImageJ, normalized to β-actin, and presented underneath the blot. Viral capsid protein (VP1) was used as a marker of CVB3 replication. Arrow denotes potential cleavage fragment. (**B** and **D**) HeLa cells were transfected with Flag-OPTN **(B)** or GFP-T6BP **(D)** for 24 h prior to CVB3 infection as above. Cell lysates were probed with either anti-Flag or anti-GFP antibody. Protein levels of T6BP were quantified as above.

Mitochondrial quality control is regulated by a selective autophagy process termed as mitophagy through the activity of autophagy receptor proteins (Chu, 2019). We previously reported that CVB3 actively targets autophagy receptor proteins, including p62, NBR1, and NDP52, through virally encoded proteinases (Shi et al., 2013, 2014; Mohamud et al., 2019). In this study, we wanted to investigate whether other receptor proteins, namely the autophagy receptors OPTN and TAX1BP1/T6BP, may also be targeted by CVB3. To this end, we performed time-course CVB3 infections in HeLa cells to monitor the protein levels of OPTN and T6BP (Figures 2A,C). In parallel, we performed time-course infections in HeLa cells overexpressing exogenous Flag-tagged OPTN (Figure 2B) or GFP-tagged T6BP (Figure 2D). We found that protein levels of endogenous as well as exogenous OPTN were stable during early phase of CVB3 infection and slightly decreased upon infection (Figures 2A,B). In contrast, CVB3 infection beyond the 5 h time point caused a marked reduction in the protein levels of both endogenous and exogenous T6BP (Figures 2C,D). Of note, Western blotting using an anti-T6BP antibody that was raised against amino acids 387–471 and an anti-GFP antibody that detects exogenous GFP-T6BP revealed an extra antibody-reactive band at ~88 kDa and ~115 kDa, respectively, suggesting possible cleavage of T6BP following CVB3 infection.

### T6BP Is Cleaved by Viral Proteinase 2A

**Figure 3:**
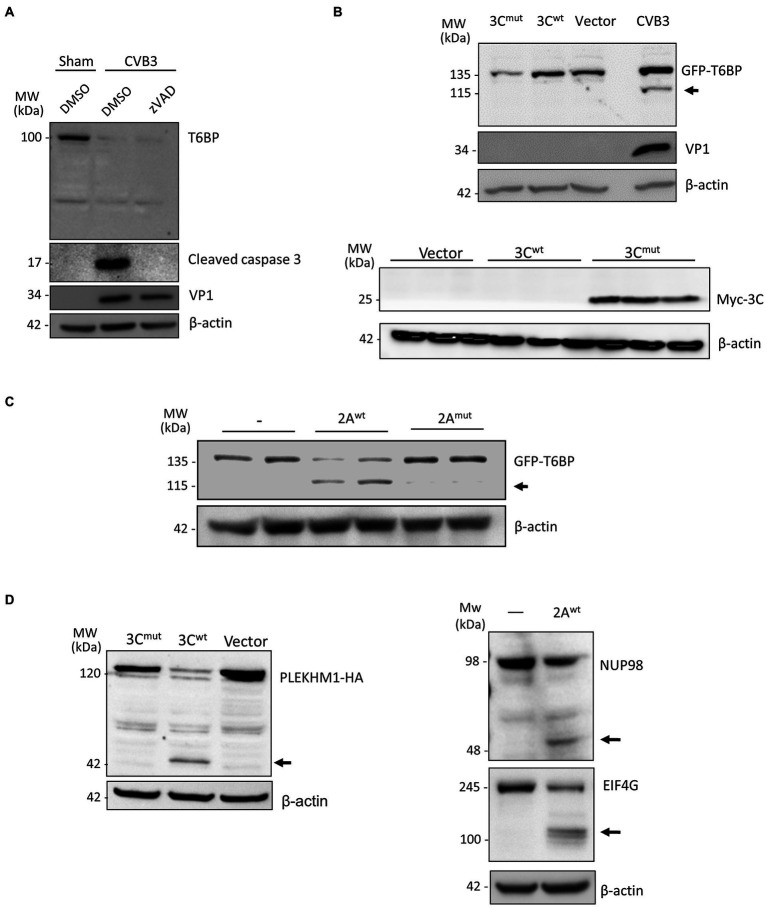
T6BP is cleaved by 2A^pro^. **(A)** HeLa cells were infected with CVB3 (MOI = 10) for 7 h in the presence of a pan-caspase inhibitor (zVAD-FMK, 50 μm) or vehicle control (DMSO). Western blot analysis was performed with anti-T6BP or anti-cleaved caspase 3 antibody. **(B)** HeLa cells were transfected with GFP-T6BP together with either catalytically inactive myc-3C^mut^, myc-wild-type 3C (3C^wt^), or control empty vector. After 24 h, cell lysates were collected and probed with anti-GFP and VP1 antibodies (upper panel) or with anti-myc antibody (bottom panel). CVB3-infected cell lysates were used as a positive control. **(C)** *In vitro* cleavage assay was performed by incubating GFP-T6BP-expressing HeLa lysates with vehicle (−), purified wild-type 2A (2A^wt^), or catalytically inactive 2A (C109A) mutant (2A^mut^). Western blot analysis was conducted with anti-GFP antibody. Arrow denotes 2A-mediated cleavage fragment. **(D)** HeLa cells expressing PLEKHM1-HA together with either 3C^mut^, 3C^wt^, or control empty vector for 24 h were harvested for Western blot analysis with anti-HA antibody (left). *In vitro* cleavage assay was performed through incubation of purified 2A^wt^ with HeLa lysates and analyzed by Western blot analysis with anti-NUP98 and anti-EIF4G antibodies. Black arrows denote cleavage fragments.

To exclude the role of host caspases in CVB3-mediated degradation of T6BP, we infected HeLa cells with CVB3 in the presence of the pan-caspase inhibitor zVAD-FMK (50 μm). The inhibition of caspase activity was unable to rescue T6BP degradation (Figure 3A). CVB3 expresses two cysteine proteinases, 2A and 3C, that primarily act to process the viral polyprotein. Co-transfection of cells with GFP-T6BP and either wild-type 3C (3C^wt^) or a catalytically inactive (C147A) mutant 3C (3C^mut^) failed to recapitulate the cleavage fragment observed following CVB3 infection (Figure 3B, upper panel). The expected band was observed at ~26 kDa for myc-3C^mut^ but not myc-3C^wt^ (Figure 3B, bottom panel), indicating an auto-cleavage at the N-terminus as previously reported (Mukherjee et al., 2011). However, *in vitro* cleavage assay using purified WT 2A (2A^wt^), but not a catalytically inactive (C109A) 2A mutant (2A^mut^), was able to generate the expected 115 kDa cleavage fragment that was previously observed following CVB3 infection (Figure 3C). The enzymatic activities of 3C^wt^ construct and recombinant 2A^wt^ were confirmed by the cleavage of previously identified substrates [i.e., PLEKHM1 for 3C^wt^ and NUP98 and EIF4G for 2A^wt^, respectively (Chau et al., 2007; Mohamud et al., 2018; Hanson et al., 2019); Figure 3D]. Collectively, CVB3 utilizes the activity of 2A^wt^, but not 3C^wt^ or host caspase, to process T6BP.

### 2A^wt^ Cleaves T6BP After G621

**Figure 4:**
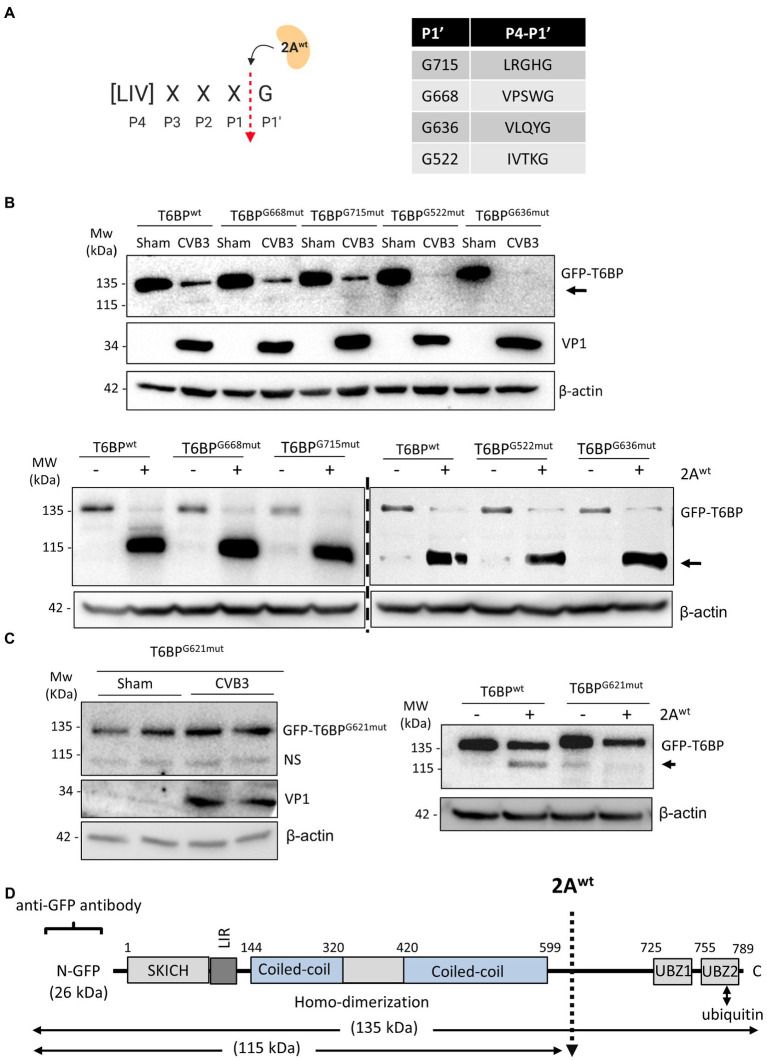
2A^wt^ cleaves T6BP after G621. **(A)** Schematic depiction of 2A^wt^ consensus cleavage site. Red arrow denotes the scissile cleavage between P1 residue and the conserved glycine (G) residue at P1′. Right, consensus sequences identified in the open reading frame of T6BP and the respective P1' G residues. **(B)** HeLa cells were transfected with either GFP-T6BP^wt^ or various mutants T6BP constructs as indicated. After 24 h, cells were sham or CVB3 infected for 7 h (upper panel) or collected for *in vitro* cleavage assay with purified 2A^wt^ (bottom panel). Western blot analysis was conducted with anti-GFP and anti-VP1 antibodies. Arrows denote T6BP cleavage fragments. **(C)** HeLa cells were transfected with either GFP-T6BP^wt^ or T6BP-G621^mut^ as indicated. After 24 h, cells were sham or CVB3 infected for 7 h (left panel; duplicates of each condition are shown) or harvested for *in vitro* cleavage assay with purified 2A^wt^ (right panel). Western blotting was performed as above. NS, non-specific band. **(D)** Schematic illustration of the structural domains of T6BP, the identified cleavage site, the antibody recognition regions, and the resulting cleavage products.

We next sought to identify the site of cleavage by 2A^wt^. To narrow down the potential cleavage sites, we referenced the known consensus cleavage sequence of 2A^wt^ (Blom et al., 1996), with the highly conserved scissile bond occurring between P1 and the glycine (G) P1′ residue (Figure 4A). Cross-referencing this consensus sequence against the T6BP open reading frame, we obtained four candidate cleavage sites at positions G668, G715, G522, and G636. Mutant fragments were subcloned into the original GFP backbone of the WT-T6BP and utilized for *in vitro* cleavage assay. Interestingly, all 4 mutants were susceptible to CVB3 and 2A^wt^-mediated cleavage, similar to WT-T6BP (Figure 4B). We therefore expanded the consensus cleavage sequence to include atypical residues at the P4 position and discovered multiple additional sites. Western blot analysis following CVB3 infection or *in vitro* cleavage assay with recombinant 2A^wt^ revealed that G621^mut^ was resistant to cleavage (Figure 4C). Collectively, these results support that viral proteinase 2A cleaves T6BP after G621 although additional cleavage sites could not be ruled out (Figure 4D). It is noted that the efficiency of 2A-mediated cleavage of GFP-T6BP in Figure 4B (bottom panel) and Figure 4C (right panel) was inconsistent, which is most likely due to the use of different batches of purified 2A proteins that have differential enzymatic activities.

### T6BP and OPTN Regulate CVB3 Infectivity

**Figure 5:**
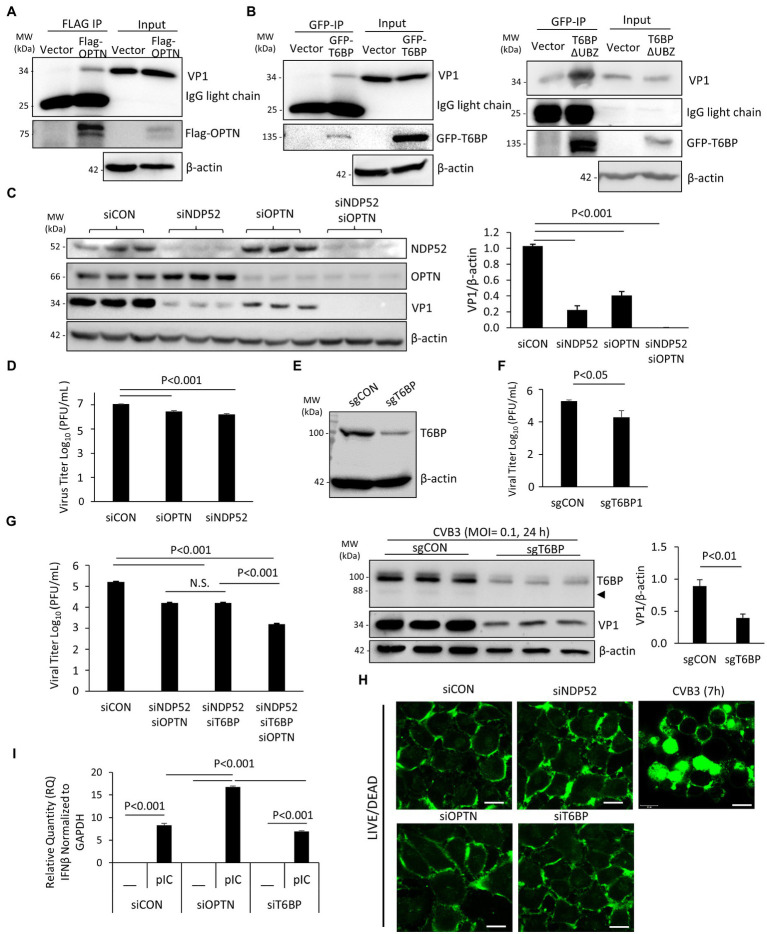
T6BP and OPTN regulate CVB3 infectivity. (**A** and **B**) HeLa cells were transfected with either 3× Flag-OPTN **(A)**, GFP-T6BP (B, left), or GFP-T6BP-ΔUBZ (B, right) for 24 h prior to CVB3 infection (MOI = 10) for additional 5 h. Cell lysates were subjected to immunoprecipitation with anti-Flag **(A)** or anti-GFP **(B)** antibodies, respectively, and analyzed by Western blotting. **(C)** HeLa cells were transiently transfected with small interfering RNA (siRNAs) targeting NDP52 (siNDP52) or/and OPTN (siOPTN), or a scramble siRNA control (siCON) as indicated for 48 h, followed by CVB3 infection (MOI = 0.1) for 24 h. Western blotting was performed to examine the protein expression of NDP52, OPTN, VP1, and β-actin. Protein levels of VP1 were quantified by densitometric analysis and presented in the bar graph (mean ± SD, *n* = 3). **(D)** HeLa cells were treated as above. Cell-associated virus titers were determined by TCID50 assay. Data are represented as mean ± SD from three replicates. **(E)** HeLa cells were transfected with constructs expressing sgRNA targeting T6BP (sgT6BP) or scramble control (sgCON) for 48 h. Knockdown efficiency was verified by Western blotting with anti-T6BP antibody. **(F)** HeLa cells were treated as above **(E)** and infected with CVB3 (MOI = 0.1) for 24 h. Cell-associated virus titers were determined by TCID50 assay. Data are representative of mean ± SD from three replicates. Corresponding lysates from parallel experiments were subjected to Western blot analysis with anti-T6BP, VP1, and β-actin antibodies (bottom panel). Protein levels of VP1 were quantified by densitometric analysis and presented in the right bar graph (mean ± SD, *n* = 3). **(G)** HeLa cells were treated with siNDP52/siOPTN, siNDP52/siT6BP, siNDP52/siOPTN/siT6BP, or siCON as indicated for 48 h, followed by CVB3 infection (MOI = 0.1) for 24 h. Cell-associated virus titers were determined by TCID50 assay. Data are representative of mean ± SD from three replicates. **(H)** HeLa cells were treated with siNDP52, siOPTN, siT6BP, or siCON for 48 h and subsequently stained with the LIVE/DEAD membrane integrity probe. Cells were fixed and visualized under confocal microscopy with an excitation wavelength of 580 nm. HeLa cells infected with CVB3 (MOI = 10) for 7 h were used as a positive control for non-viable cells. Scale bar = 20 μm. **(I)** HeLa cells were treated with siOPTN, siT6BP, or siCON for 48 h and subsequently transfected with poly I:C (pIC, 1μg/ml) for another 8h. Cells were collected for RNA extraction, and qRT-PCR was conducted to determine the mRNA levels of IFNβ (mean ± SD, *n* = 3).

It was previously demonstrated that p62 and NDP52 can interact with viral capsid protein but differentially regulate viral replication through their distinct actions on the MAVS-mediated antiviral type I IFN signaling (Mohamud et al., 2019). Here, we examined the role of OPTN and T6BP in viral replication. Firstly, we tested whether these autophagy receptors could interact with viral capsid protein, thereby targeting viral particles for virophagy. HeLa cells were transfected with Flag-OPTN, GFP-T6BP, or vector control prior to CVB3 infection. Immunoprecipitation with anti-Flag or anti-GFP antibody revealed the pull-down of viral capsid protein VP1 in cells expressing Flag-OPTN or GFP-T6BP, but at a relatively low efficacy, suggesting limited binding affinity (Figures 5A,B). Interestingly, we observed that the cleaved fragment of T6BP (T6BP-ΔUBZ) retained the ability of full-length T6BP to bind with VP1, suggesting that this interaction is ubiquitin independent (Figure 5B). We next investigated the impacts of OPTN and T6BP deletion on viral replication. Using siRNA, OPTN and NDP52 were genetically silenced in HeLa cells either individually or in combination. Following 48 h transfection, cells were infected with CVB3 (MOI = 0.1, 24 h), followed by Western blot analysis and quantitation of cell-associated viruses. We found that knockdown of NDP52 and/or OPTN led to significant attenuation of viral protein production (Figure 5C) and reduction in cell-associated viral titers (Figure 5D). To assess the role of T6BP in viral replication, we also designed sgRNA targeting exon 2 of T6BP and transfected HeLa cells with a construct expressing sgRNA and Cas9. Protein knockdown was confirmed with anti-T6BP antibody following 48 h transfection (Figure 5E). In parallel, cells were infected with CVB3 at an MOI = 0.1 for 24 h and viral titers were assessed. Compared to control, cells expressing sgRNA targeting T6BP had significantly reduced viral titers and decreased levels of viral capsid protein (Figure 5F). Due to the lower MOI of infection used in this assay, the predominant form of T6BP was the full length. Consistent with these observations, combination treatments of double knockdown with siNDP52/siOPTN or siNDP52/siT6BP, or triple silencing with siNDP52/siOPTN/siT6BP, resulted in progressively reduced viral titers (Figure 5G). Cell viability following siRNA treatment was assessed using the membrane integrity LIVE/DEAD dye, and no significant alterations in membrane integrity following treatment with various siRNAs were observed (Figure 5H). Cells infected with CVB3 at 7 h were used as a control, showing disrupted membrane integrity and intracellular penetration of the LIVE/DEAD dye (Figure 5H). To investigate the pro-viral mechanisms of OPTN and T6BP, we tested their contributions to the antiviral type I IFN pathway. HeLa cells were treated with siRNA targeting OPTN, T6BP, or scrambled control siRNA for 48 h, followed by stimulation with the synthetic double-stranded RNA analog, poly I:C. Robust stimulation of IFNβ gene expression was observed in poly I:C-treated cells compared to untreated cells. Of note, we found that IFNβ mRNA level was significantly upregulated in siOPTN-treated cells compared to control or siT6BP-treated cells (Figure 5I), suggesting a pro-viral mechanism for OPTN through suppressing the expression of IFNβ. Taken together, our results indicate that, similar to NDP52 (Mohamud et al., 2019), both OPTN and T6BP play a pro-viral function during CVB3 infection.

### Cleavage Fragments of NDP52 and T6BP Lose the Binding Affinity of Native Proteins and Fail to Co-Localize With Mitochondria

**Figure 6:**
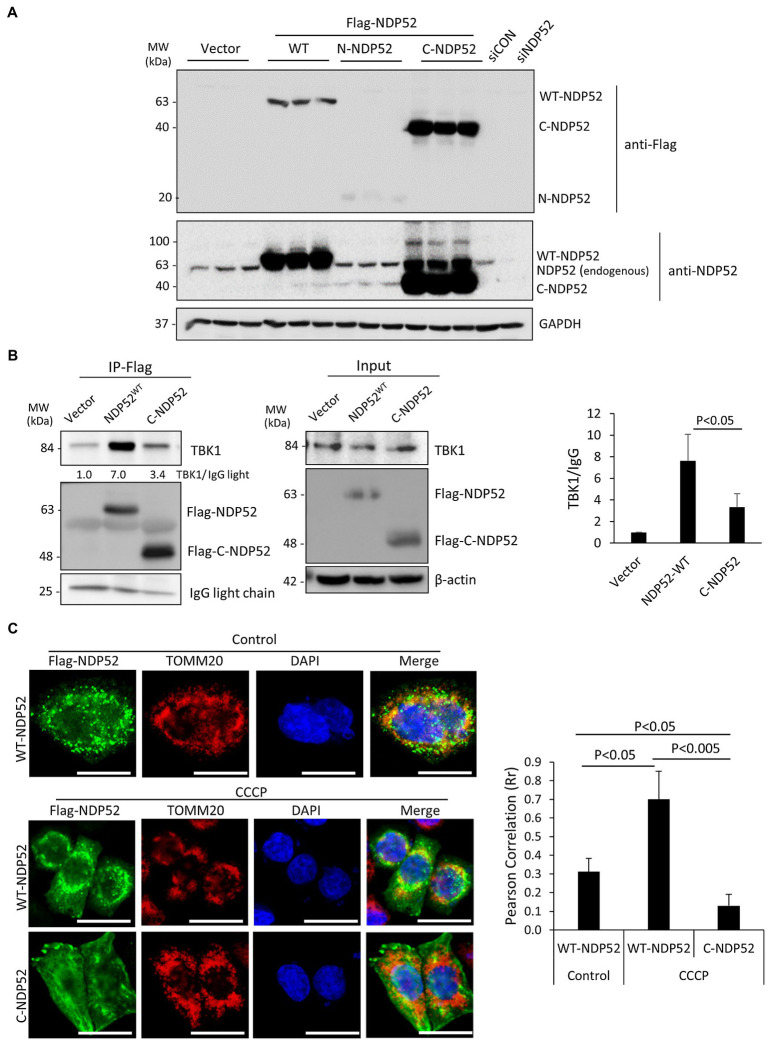
The C-terminal NDP52 cleavage fragment shows the reduced TBK1 binding affinity and impaired mitochondrial localization. **(A)** HeLa cells were transfected with various Flag-tagged NDP52 constructs as indicated for 24 h. Cell lysates were analyzed by Western blotting with anti-Flag and anti-NDP52 antibodies. **(B)** HeLa cells were transfected with empty vector, Flag-NDP52^wt^, or Flag-C-NDP52 for 24 h. Cell lysates were subjected to immunoprecipitation with anti-Flag antibody and analyzed by Western blotting. The amount of TBK1 pull-down was normalized to anti-Flag IgG light chain and presented in the bar plot (mean ± SD, *n* = 3 independent experiments). **(C)** HeLa cells were transfected with either Flag-NDP52^wt^ or Flag-C-NDP52 for 24 h. Cells were then treated with either vehicle or CCCP (10 μm) for 6 h, followed by immunostaining with anti-Flag (green) for NDP52 and anti-TOMM20 (red) for mitochondria. Co-localization was assessed using NIH ImageJ software and presented as Pearson’s correlation (Rr ± SD, *n* > 30 cells) in the right panel. Scale bar = 20 μm.

Despite the demonstrated role of NDP52 in CVB3 replication (Mohamud et al., 2019), the consequence of NDP52 cleavage to host selective autophagy remains unclear. Based on the identified cleavage site (after G139) on NDP52 (Mohamud et al., 2019), we generated three Flag-tagged NDP52 constructs (i.e., WT-, N-, and C-NDP52). After transient transfection, Western blotting demonstrated abundant expression of C-NDP52 (Figure 6A). However, it was noted that protein level of N-NDP52 was very low after transfection similar to our early observation (Mohamud et al., 2019), suggesting possible instability of this fragment (139 amino acids) that is small and likely undergoes cellular protease-mediated degradation (Fung et al., 2015; Mohamud et al., 2019). We therefore focused our following study on the C-NDP52.

The function of NDP52 is post-translationally regulated through direct phosphorylation by the autophagy receptor kinase TBK1. It was established that recruitment of TBK1 to NDP52 requires the N-terminal SKICH domain (Thurston et al., 2009). To investigate whether CVB3-mediated loss of the N-terminal SKICH leads to disrupted regulatory dynamics with TBK1, we tested the binding capacity of full-length NDP52 and C-NDP52 with TBK1. Immunoprecipitation studies revealed that compared to full-length NDP52, C-NDP52 had significantly reduced the interaction with TBK1 (Figure 6B). Of note, C-NDP52 still exhibited some residual interaction with TBK1, which may be partially explained by its intact coiled-coil domain that allows C-NDP52 to complex with endogenous NDP52.

To assess whether C-NDP52 could be recruited to mitochondria, we utilized the mitochondrial uncoupler, carbonyl cyanide m-chlorophenylhydrazine (CCCP) to induce depolarization of mitochondria in cells expressing either full-length NDP52 or C-NDP52. As expected, the mitochondrial marker TOMM20 formed distinct punctate foci following CCCP treatment. Full-length NDP52 was able to be recruited to the depolarized mitochondrial foci (Rr = 0.70 ± 0.15); however, C-NDP52 remained aberrantly distributed within the cells (Rr = 0.13 ± 0.06; Figure 6C).

**Figure 7:**
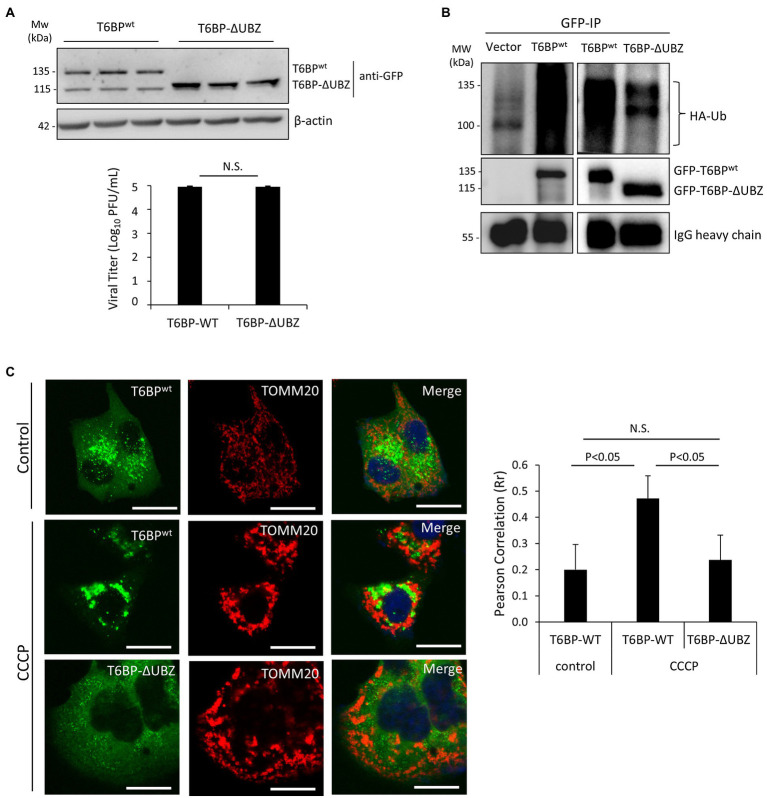
The N-terminal T6BP fragment has the reduced binding affinity with ubiquitin and fails to be effectively recruited to mitochondria. **(A)** HeLa cells were transiently transfected with GFP-T6BP^wt^ or GFP-T6BP-ΔUBZ for 24 h. Cells were subsequently infected with CVB3 (MOI = 0.1) for 24 h. Lysates were harvested for Western blot analysis with anti-GFP and β-actin antibodies. Supernatants were harvested for quantitation of viral titers and presented in the bar graph (mean ± SD, *n* = 3). **(B)** HeLa cells were transfected with HA-ubiquitin, together with either empty vector, GFP-T6BP^wt^, or GFP-T6BP-ΔUBZ for 24 h. Cell lysates were subjected to immunoprecipitation with anti-GFP antibody, followed by Western blot analysis of the interaction between WT-T6BP or T6BP-ΔUBZ and ubiquitin using anti-HA and anti-GFP antibodies. **(C)** HeLa cells were transfected with either GFP-T6BP^wt^ or GFP-T6BP-ΔUBZ for 24 h. Cells were then treated with either vehicle or CCCP (10 μm) for 6 h. Co-localization of T6BP and mitochondrial marker TOMM20 was assessed using NIH ImageJ software and presented as Pearson’s correlation (Rr ± SD, *n* > 30 cells) in the right panel. Scale bar = 20 μm.

We next investigated the effects of the cleavage fragments of T6BP on viral propagation. Given that the C-terminal fragment of T6BP is small (168 amino acids) and easily subjected to degradation by cellular proteases, we decided to focus on the N-terminal cleaved fragment of T6BP that lacks the C-terminal ubiquitin-associated domain (designated ΔUBZ). HeLa cells were transfected with exogenous GFP-T6BP^wt^ or GFP-T6BP-ΔUBZ for 24 h, followed by prolonged CVB3 infection (24 h) at a reduced MOI of 0.1 to discern subtle differences in propagation that would otherwise be masked during high MOI infections. Nevertheless, no significant differences in viral titers were observed between ectopic expression of GFP-T6BP^wt^ and GFP-T6BP-ΔUBZ, suggesting that cleavage of T6BP likely plays a minor role in viral propagation (Figure 7A).

To explore the consequences of T6BP cleavage for the host, we proceeded to test whether this cleavage disrupts its function as an autophagy receptor protein. Co-immunoprecipitation was performed by transfecting cells with HA-labeled ubiquitin construct and either full-length GFP-T6BP^wt^ or GFP-T6BP-∆UBZ. As anticipated, the ∆UBZ fragment of T6BP had impaired capacity to associate with ubiquitin (Figure 7B). To assess whether cleaved T6BP could be recruited to mitochondria, cells were transfected with either full-length GFP-T6BP^wt^ or GFP-T6BP-∆UBZ. Following 24 h, cells were treated with CCCP to induce mitochondrial depolarization and immunostained with the mitochondrial marker TOMM20. Compared to the full-length T6BP, the cleaved form lacking the UBZ domain had significantly impaired capacity to co-localize with mitochondria (0.47 ± 0.08 vs. 0.23 ± 0.09; Figure 7C). Collectively, these results support that CVB3-mediated cleavage fragments of NDP52 and T6BP have impaired adaptor functions.

## Discussion

EVs are known to hijack the host autophagy pathway to facilitate efficient replication, but the harmful consequences of autophagy dysregulation for cellular homeostasis are poorly understood. As a quality control process, selective autophagy acts to recycle damaged organelles, including mitochondria, in order to maintain cellular function. Consequently, dysregulated autophagy is a hallmark of various neurodegenerative diseases, including Parkinson’s disease, frontotemporal dementia, and amyotrophic lateral sclerosis (Chung et al., 2018).

The mitochondrial quality control process of mitophagy is mediated by specific autophagy receptors, such as OPTN, NDP52, and T6BP (Lazarou et al., 2015), while previous findings also implicated p62 in mitochondrial clustering (Geisler et al., 2010), but not the close homologue NBR1 (Shi et al., 2014). Interestingly, EVs can directly target autophagy receptors, such as p62, and NBR1 to evade virophagy efforts by the host (Shi et al., 2013, 2014). In particular, CVB3 utilizes virus-encoded proteinases 2A and 3C to cleave p62 and NBR1 into dominant negative fragments that ultimately disrupt adaptor function. Additionally, these fragments compete with native autophagy receptors for ubiquitin and autophagy binding. The current study demonstrates that other autophagy receptors, including NDP52 and T6BP, can also be targeted by viral proteinases. In addition to facilitating viral replication through attenuation of type I IFN signaling (Mohamud et al., 2019), as a result of NDP52 cleavage, we found in this study that the C-NDP52 product fails to be recruited to depolarized mitochondria, which can partially be explained by disrupted interaction with the regulatory kinase TBK1. Due to differential association with mitochondria, these findings suggest that WT-NDP52 and C-NDP52 may attenuate MAVS-mediated type I IFN response through distinct mechanisms. We also showed that CVB3-induced cleavage of T6BP results in the truncation of the UBZ domain, leading to disrupted ubiquitin association and impaired localization to depolarized mitochondria. As the N-terminal cleavage fragment of T6BP contains an intact LIR domain (Figure 4D), it is plausible to expect that the truncated form of T6BP that retains LC3 binding affinity may recruit to autophagic sites. However, the diminished ubiquitin association of cleaved T6BP suggests a likely impairment in the capacity to recruit ubiquitinated cargo.

The role of autophagy receptors in EV replication appears to be multifaceted. Genetic silencing of NDP52, OPTN, and T6BP resulted in the impairment of CVB3 replication, which may partially be explained by the role of NDP52 and T6BP as negative regulators of antiviral type I IFN signaling (Parvatiyar et al., 2010; Jin et al., 2017). The current study suggests that OPTN may also support CVB3 replication by suppressing type I IFN signaling (Figure 5I) although this was not observed for T6BP. Both OPTN and T6BP demonstrated a capacity to interact with viral capsid structural protein; however, the efficiency of this interaction is unclear. It was previously demonstrated that VP1 associates with ubiquitin that may act as a bridging factor for multiple autophagy receptors, including p62 and NDP52. Of note, the loss of the UBZ domain of T6BP did not preclude its interaction with VP1, suggesting that the interaction with this particular receptor is ubiquitin independent. Alternatively, exogenous T6BP-ΔUBZ through its coiled-coil domain (Ling and Goeddel, 2000) may still be able to complex with endogenous T6BP that retains its ubiquitin-binding capacity. Interestingly, ectopic expression of T6BP or T6BP-ΔUBZ showed comparable levels of viral titer despite T6BP-ΔUBZ demonstrating an impaired capacity to interact with ubiquitin and depolarized mitochondria. The precise mechanisms by which T6BP favors CVB3 propagation remain unclear, but insights from the current study support a growing understanding that viral proteinases cleave autophagy receptors to impair their intended function in the host cells. In particular, T6BP is implicated in the negative regulation of NF-κB signaling through its regulatory interactions with K63-ubiquitin-modified proteins, such as receptor-interacting protein 1 and tumor necrosis factor receptor-associated factor 6 (Verstrepen et al., 2011). Therefore, removal of the UBZ domain of T6BP and subsequent dysregulation of NF-κB signaling may underscore the pathogenic exacerbation of the host inflammatory response.

The observation that some autophagy receptors facilitate efficient viral replication prior to their processing by viral proteinases may suggest a time-dependent strategy by EVs to usurp autophagy receptors during early replication while dispensing them *via* viral proteinases during late infection. In this regard, EVs can evade the virophagy capacity of autophagy receptors as they continue to propagate. In summary, our findings suggest that EVs may actively target autophagy receptors to facilitate efficient replication, evade virophagy, and ultimately impair selective autophagy.

## Data Availability Statement

The original contributions presented in the study are included in the article/supplementary material, further inquiries can be directed to the corresponding author.

## Author Contributions

YM and HL designed the experiments. YM, YX, HLi, CN, and AB conducted the experiments and analyzed the data. YM and HLu wrote the manuscript. All authors contributed to the article and approved the submitted version.

### Conflict of Interest

The authors declare that the research was conducted in the absence of any commercial or financial relationships that could be construed as a potential conflict of interest.
